# SARS-CoV-2 Omicron infection augments the magnitude and durability of systemic and mucosal immunity in triple-dose CoronaVac recipients

**DOI:** 10.1128/mbio.02407-23

**Published:** 2024-03-08

**Authors:** Yuxin Chen, Tiantian Zhao, Lin Chen, Guozhi Jiang, Yu Geng, Wanting Li, Shengxia Yin, Xin Tong, Yue Tao, Jun Ni, Qiuhan Lu, Mingzhe Ning, Chao Wu

**Affiliations:** 1 Department of Laboratory Medicine, Nanjing Drum Tower Hospital, The Affiliated Hospital of Medical School, Nanjing University, Nanjing, Jiangsu, China; 2 Institute of Viruses and Infectious Diseases, Nanjing University, Nanjing, Jiangsu, China; 3 Department of Infectious Diseases, Nanjing Drum Tower Hospital Clinical College of Nanjing University of Chinese Medicine, Nanjing, Jiangsu, China; 4 Department of Laboratory Medicine, Nanjing Drum Tower Hospital Clinical College of Nanjing Medical University, Nanjing, Jiangsu, China; 5 School of Public Health (Shenzhen), Shenzhen Campus of Sun Yat-sen University, Shenzhen, Guangdong, China; 6 School of Public Health (Shenzhen), Sun Yat-sen University, Guangzhou, Guangdong, China; 7 Department of Infectious Diseases, Nanjing Drum Tower Hospital Clinical College of Xuzhou Medical University, Nanjing, Jiangsu, China; Monash University, Mount Waverley, Australia

**Keywords:** Omicron BA.5, CoronaVac, IgA, B cells, T cells, immune imprinting

## Abstract

The inactivated whole-virion vaccine, CoronaVac, is one of the most widely used coronavirus disease 2019 (COVID-19) vaccines worldwide. There is a paucity of data indicating the durability of the immune response and the impact of immune imprinting induced by CoronaVac upon Omicron infection. In this prospective cohort study, 41 recipients of triple-dose CoronaVac and 14 unvaccinated individuals were recruited. We comprehensively profiled adaptive immune parameters in both groups, including spike-specific immunoglobulin (Ig) G and IgA titers, neutralizing activity, B cells, circulating follicular helper T (cTfh) cells, CD4^+^ and CD8^+^ T cells, and their memory subpopulations at 12 months after the third booster dose and at 4 and 20 weeks after Omicron BA.5 infection. Twelve months after the third CoronaVac vaccination, spike-specific antibodies and cellular responses were detectable in most vaccinated individuals. BA.5 infection significantly augmented the magnitude, cross-reactivity, and durability of serum neutralization activities, Fc-mediated phagocytosis, nasal spike-specific IgA responses, memory B cells, activated cTfh cells, memory CD4^+^ T cells, and memory CD8^+^ T cells for both the ancestral strain and Omicron subvariants, compared to unvaccinated individuals. Notably, the increase in BA.5-specific immunity after breakthrough infection was consistently comparable to or higher than that of the ancestral strain, suggesting no evidence of immune imprinting. Immune landscape analyses showed that vaccinated individuals have better synchronization of multiple immune components than unvaccinated individuals upon heterologous infection. Our data provide detailed insight into the protective role of the inactivated COVID-19 vaccine in shaping humoral and cellular immunity to Omicron infection.

**IMPORTANCE:**

There is a paucity of data indicating the durability of the immune response and the impact of immune imprinting induced by CoronaVac upon Omicron breakthrough infection. In this prospective cohort study, the anti-severe acute respiratory syndrome coronavirus 2 adaptive responses were analyzed before and after the Omicron BA.5 infection. Our data provide detailed insight into the protective role of the inactivated COVID-19 vaccine in shaping humoral and cellular immune responses to heterologous Omicron infection.

**CLINICAL TRIAL:**

This study is registered with ClinicalTrials.gov as NCT05680896.

## INTRODUCTION

Three years after the coronavirus disease 2019 (COVID-19) outbreak caused by severe acute respiratory syndrome coronavirus 2 (SARS-CoV-2), remarkable collaborative efforts have been made to mitigate the pandemic. The worldwide COVID-19 vaccination campaign demonstrated outstanding effects in reducing the severity of SARS-CoV-2 infection (1, 2). CoronaVac, a whole-virion inactivated vaccine produced by Sinovac, is the most widely offered COVID-19 vaccine globally. Although the diminished immunity and emergence of new viral variants have fueled an increase in the frequency of breakthrough infections, a real-world efficacy analysis in China showed that the protection efficacy of the triple-dose inactivated vaccine against BA.2 infection was 74% against pneumonia and 93% against severe COVID-19 (3).

Understanding the underlying immunological mechanism accounting for this vaccine-derived protection is not only crucial for next-generation COVID-19 vaccine optimization but also has important implications for future pandemic preparedness. In a prospective vaccine cohort, we previously analyzed the longitudinal immune responses elicited by the triple-dose inactivated COVID-19 vaccine for up to 2 months, including serum-neutralizing antibody activity and B and T cell immune memory responses (4–7). Since the adjustment of the public health policy for containing COVID-19 in China in December 2022, the subsequent SARS-CoV-2 Omicron variant wave resulted in large-scale breakthrough infection. In this study, we aimed to address two equally important issues. First, we examined whether CoronaVac-induced immune memory could still be effectively recalled up to 12 months after the third booster dose. The Omicron variant is associated with a marked capability to evade humoral immunity resulting from either prior natural infection or vaccination (8, 9). There are rising concerns regarding the immune imprinting induced by ancestral strain-based vaccinations, which might compromise the antibody response to Omicron-based boosters. Therefore, we also attempted to investigate whether the immune responses elicited by triple-dose CoronaVac had any beneficial role in the anti-viral responses to subsequent Omicron infection.

In this study, we investigated these key questions by measuring SARS-CoV-2-specific dynamic immune trajectories, including antibody responses, Fc-mediated effector function, neutralization capacity, and cellular responses after Omicron BA.5 infection among recipients of triple-dose CoronaVac and unvaccinated individuals. Our data provide detailed insight into the favorable role of pre-existing immune responses elicited by triple-dose CoronaVac, which are capable of potently recalling SARS-CoV-2 immunity with cross-recognition to Omicron variants.

## RESULTS

### Study cohort and study design

**Fig 1:**
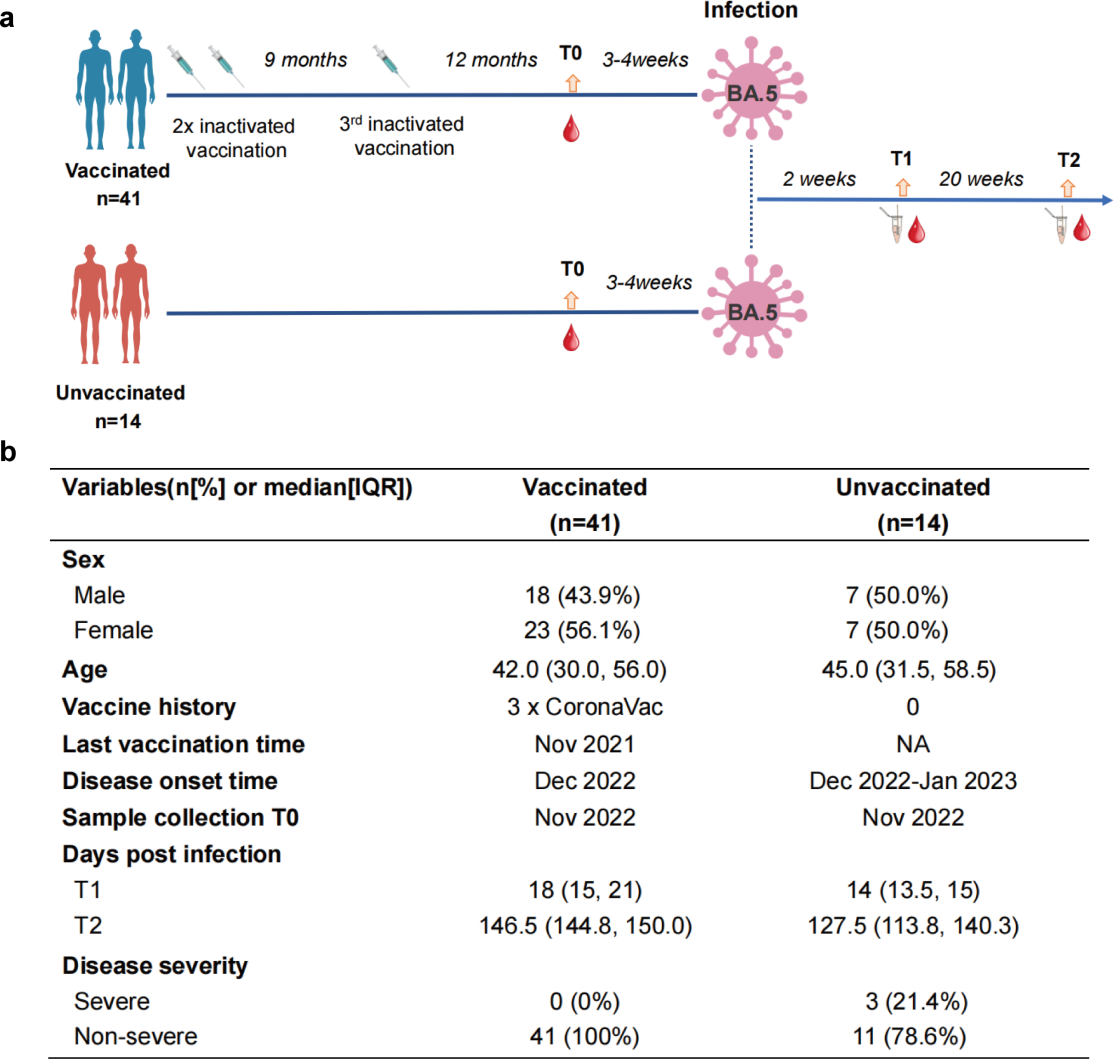
Study design and demographic characteristics of our cohort. (a) Study design of vaccinated individuals and unvaccinated individuals with Omicron BA.5 infection. Vaccination, infection, and blood draw timeline were also noted. (b) The demographic and clinical characteristics of the study cohort.

We recruited a total of 55 participants, including 41 recipients of triple-dose CoronaVac and 14 individuals who were not vaccinated against COVID-19 (Fig. 1a). Omicron BA.5 infection was confirmed in all individuals by next-generation sequencing. The demographic and clinical characteristics of the cohort are summarized in Fig. 1b. The median age in the vaccinated group is 42.0 [95% confidence interval (CI) 30.0, 56.0], and the unvaccinated group is 45.0 (95% CI 31.5, 58.5). Out of 14 unvaccinated individuals, 3 (21.4%), but none of the vaccinated individuals, developed severe COVID-19 disease. Sampling at 12 months after the third-booster dose (T0), as well as at 2 weeks (T1) and 20 weeks (T2) after SARS-CoV-2 infection, allowed the immune trajectories to be measured to assess the establishment and maintenance of hybrid immunity. Simultaneous serum, peripheral blood mononuclear cell, and nasal swab samples were collected from each of the participants. The samples were subjected to a detailed analysis for both serological and cellular immune responses to SARS-CoV-2 antigens. The serum samples were tested for specific antibody titers using the enzyme-linked immunosorbent assay (ELISA) and neutralization activities against the ancestral, Delta, and Omicron BA.5 variants. We analyzed Fc-mediated phagocytosis activity to evaluate any functional differences in the antibody responses among vaccinated and unvaccinated individuals after BA.5 infection.

### Breakthrough infection enhanced the magnitude and durability of humoral antibody responses

**Fig 2:**
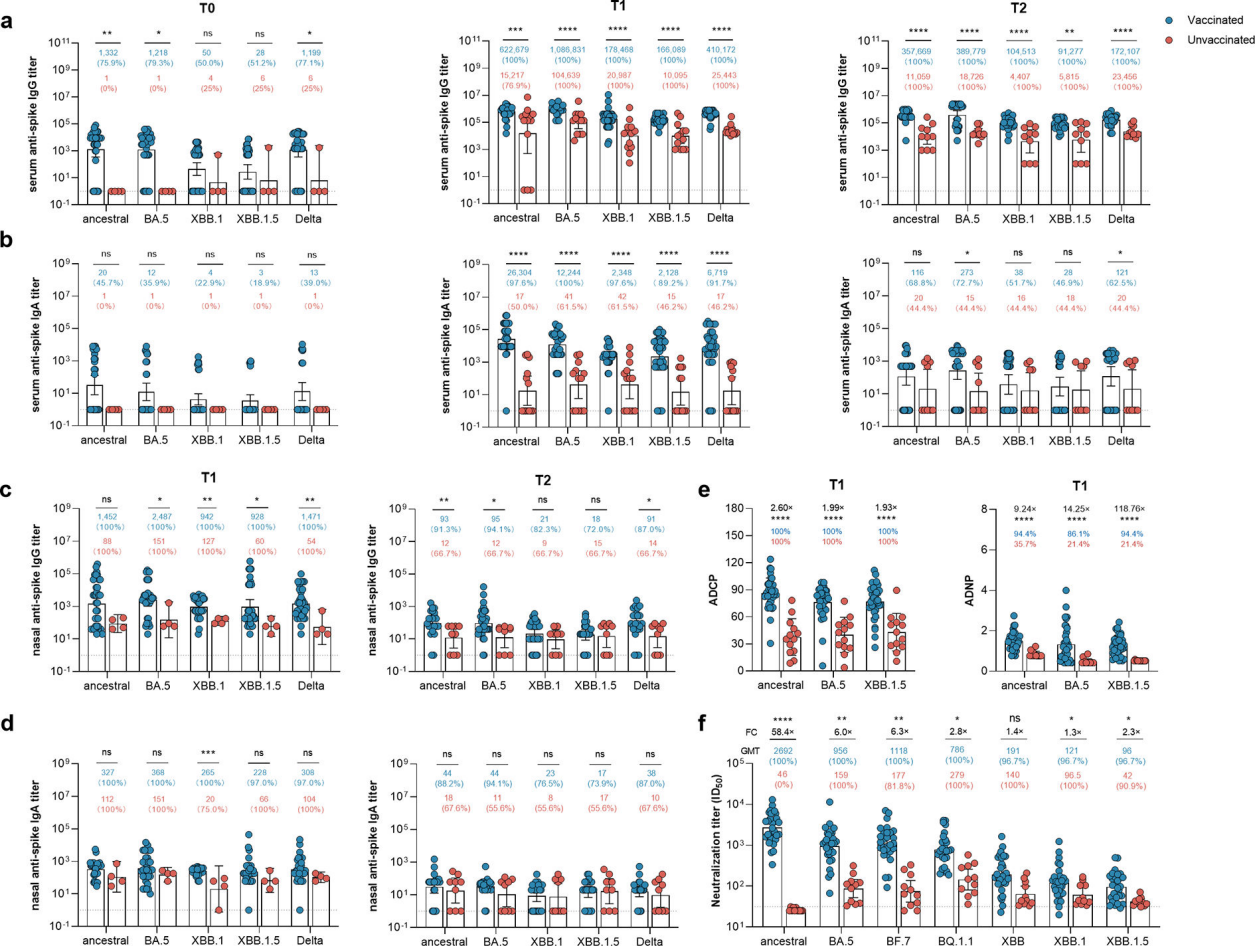
Omicron BA.5 infection enhanced serum and mucosal humoral response in triple CoronaVac vaccinated individuals. (a–d) Enzyme-linked immunosorbent assay measurement for serum anti-spike IgG titer (a), serum anti-spike IgA titer (b), nasal anti-spike IgG titer (c), and nasal anti-spike IgA titer (d) at different time points, including 12 month after the third dose vaccination (**T0**), 2 weeks (**T1**), and 20 weeks (**T2**) post-BA.5 infection. (e) Antibody-dependent cellular phagocytosis (ADCP) and antibody-dependent neutrophil phagocytosis (ADNP) specific to spike protein of ancestral, BA.5, and XBB.1.5 at T1 were also analyzed. (f) Serum titers that achieved 50% pseudovirus neutralization (ID50) of seven SARS-CoV-2 variants, including ancestral strain, BA.5, BF.7, BQ1.1, XBB, XBB.1, and XBB.1.5, in vaccinated and unvaccinated individuals after BA.5 infection at T1 time point. Blue dots represent previous triple CoronaVac vaccinated individuals, and red dots represent unvaccinated individuals. Dotted lines indicate the lower limit of detection for the assay. Data points on the bar graph represent individual titer and the line indicates geometric mean titer (GMT). GMTs of each group were noted on the top of each bar. Statistics were calculated using the Mann-Whitney *U* test for single comparison variables between groups. *P < 0.05; **P < 0.01; ***P < 0.001; ****P < 0.0001; and ns, no significant difference.

Antibodies are considered as the correlates of protection against SARS-CoV-2 infection (10). Therefore, we investigated the magnitude of variant-specific immunoglobulin (Ig)G and IgA binding in the serum. First, the binding of IgG and IgA with ancestral, Delta, BA.5, XBB.1, and XBB.1.5 was assessed by analyzing the serum collected before infection and at 2 and 20 weeks after SARS-CoV-2 infection (Fig. 2a and b).

Baseline sera at T0 exhibited an ancestral spike-specific binding geometric median titer (GMT) of 1,332 (95% confidence interval 358–4,959), while the GMT specific to BA.5, XBB.1, and XBB.1.5 was 1,218 (95% CI 365–4,064), 50 (95% CI 17–149), and 28 (95% CI 8–95), respectively (Fig. 2a).

The convalescent serum from vaccinated individuals at T1 showed an increment of 467-fold for anti-ancestral spike IgG, with a GMT of 622,679 (95% CI 433,486–894,445). The respective fold elevation in anti-BA.5, anti-XBB.1, and anti-XBB.1.5 spike IgG titers was 892-fold (1,086,831, 95% CI 820,495–1,439,620), 3,569-fold (178,468, 95% CI 105,510–301,876), and 5,932-fold (166,089, 95% CI 129,837–212,463), respectively. Compared to unvaccinated individuals, vaccinated individuals exhibited a considerably higher ancestral spike-specific IgG titer (622,679 vs 15,217, *P* = 0.006) and BA.5 spike-specific IgG titer (1,086,831 vs. 104,639, *P* < 0.0001). Similar patterns were also observed for the XBB.1, XBB.1.5, and Delta spike-specific IgG titers.

We assessed the durability of the binding antibody titers at T2 (20 weeks after BA.5 infection). Compared with the unvaccinated controls, vaccinated individuals retained a significantly greater anti-spike IgG titer not only specific to the ancestral spike (11,059 vs 357,669, *P* < 0.0001) but also to the BA.5 spike (18,726 vs 389,779, *P* < 0.0001), XBB.1 spike (4,407 vs 104,513, *P* < 0.0001), XBB.1.5 spike (5,815 vs 91,277, *P* = 0.003), and Delta spike (23,456 vs 172,107, *P* < 0.0001). Meanwhile, dynamic serological SARS-CoV-2-specific IgA responses were also detected (Fig. 2b). After BA.5 infection, 40 of 41 fully vaccinated individuals (97.6%) were IgA-positive for the ancestral spike, and 100%, 97.6%, and 89.2% were IgA-positive for the BA.5, XBB.1, and XBB.1.5 spikes, respectively. Of the 14 unvaccinated individuals, 7 (50%) were IgA-positive for the ancestral spike, and 61.5%, 61.5%, and 46.2% were IgA-positive for the BA.5, XBB.1, and XBB.1.5 spikes, respectively. Compared to T0, BA.5 infection induced strong recall responses in vaccinated individuals, leading to 1,315-fold, 1,020-fold, 587-fold, and 709-fold increased IgA titers specific to the ancestral, BA.5, XBB.1, and XBB.1.5 strains, respectively, at T1. Consistently, vaccinated individuals showed significant enhancement of spike-specific IgA responses across the tested variants compared with unvaccinated individuals (all *P* < 0.0001). Vaccinated individuals had significantly higher BA.5 spike and Delta spike IgA responses at T2 (*P* = 0.018 and *P* = 0.032, respectively). Similar trends were also noted for the IgA titers specific to the ancestral, XBB.1, and XBB.1.5 spikes but without significant differences. Collectively, our data suggest that a prior history of vaccination facilitates antibody recall, resulting in the boosted breadth and durability of SARS-CoV-2-specific IgG and IgA responses.

### Triple-dose CoronaVac expanded nasal IgA and IgG antibody titers after acquired BA.5 infection

Recent evidence has demonstrated that mucosal immunity plays a pivotal role in respiratory viral infection, and ancestral SARS-CoV-2 spike-specific mucosal immunity is protective against Omicron infection (11). To this end, nasal IgG responses were detected with substantially elevated ancestral (1,452 vs 88, *P* > 0.05), BA.5 (2,487 vs 151, *P* = 0.017), and XBB.1.5 (928 vs. 60, *P* = 0.014) IgA responses (Fig. 2c). A similar trend was also observed in the vaccinated group compared to that in the unvaccinated group, with higher levels of nasal anti-spike IgA titer specific to BA.5 (368 vs 151, *P* > 0.05), Delta (308 vs 104, *P* > 0.05), and XBB.1 (265 vs 20, *P* = 0.0001) (Fig. 2d). However, such differences were less obvious at T2. Our data suggest that although most intramuscularly injected COVID-19 vaccines induced minimal anti-spike IgA responses, breakthrough infection via the mucosal route synergistically improved both systemic and mucosal anti-viral immunity, but the mucosal IgA response appeared to wane within 20 weeks.

### Breakthrough infection induced stronger antibody-dependent cellular and neutrophil phagocytosis than natural BA.5 infection

Fc-mediated effector functions have been demonstrated to reduce the severity of disease rather than reducing transmission; therefore, they might play a role in vaccine-attenuated disease (12). We recently reported that triple-dose CoronaVac could induce potent antibody-dependent cellular phagocytosis (ADCP) and antibody-dependent neutrophil phagocytosis (ADNP) (7). The data show that the vaccinated group had substantially elevated ADCP and ADNP compared with unvaccinated controls (Fig. 2e). Specifically, plasma samples from triple-dose CoronaVac recipients showed higher ADCP responses specific to the ancestral (2.60-fold higher, *P* < 0.0001), BA.5 (1.99-fold higher, *P* < 0.0001), and XBB.1.5 (1.93-fold higher, *P* < 0.0001) spikes than unvaccinated controls. Minimal ADNP activity was detected among unvaccinated individuals, while the vaccinated individuals had considerably enhanced ancestral (9.24-fold higher, *P* < 0.0001), BA.5 (14.25-fold higher, *P* < 0.0001), and XBB.1.5 (118.76-fold higher, *P* < 0.0001) spike ADNP activity.

### Breakthrough infection enhanced the potency of neutralization activity against BA.5, XBB.1, and XBB.1.5

Omicron subvariants, especially XBB subvariants, are considered to be highly resistant to vaccines and infection-induced humoral immunity (13, 14). Using the pseudovirus neutralization assay, the serum neutralization capability against the most tested variants was significantly increased in vaccinated individuals compared with unvaccinated controls. The geometric mean 50% neutralizing antibody titers ID_50_ GMTs) for the ancestral, BA.5, BF.7, BQ1.1, XBB, XBB.1, and XBB.1.5 strains in the vaccinated group were 2,692, 956, 1,118, 786, 191, 121, and 96, respectively. The ID_50_ GMTs of the unvaccinated group against the ancestral (46; 58.4-fold, *P* < 0.0001), BA.5 (159; 6.0-fold, *P* = 0.002), BF.7 (177; 6.3-fold, *P* = 0.003), BQ1.1 (279; 2.8-fold, *P* = 0.032), XBB (140; 1.4-fold, *P* = 0.486), XBB.1 (96.5; 1.3-fold, *P* = 0.044), and XBB.1.5 (42; 2.3-fold, *P* = 0.023) strains were significantly compromised (Fig. 2f). The largest difference was observed for neutralization activity against the ancestral strain, whereas the smallest difference was observed for the XBB.1 strain between vaccinated and unvaccinated individuals. The data suggest that BA.5 neutralization activity was effectively boosted once the virus had invaded the oropharynx among vaccinated individuals who received the ancestral strain-based CoronaVac. Furthermore, the immune escape of the XBB subvariants from Omicron convalescent serum was partly restored by hybrid immunity.

### BA.5 infection enhanced cross-reactive spike-specific B cell responses that persisted for up to 20 weeks

**Fig 3:**
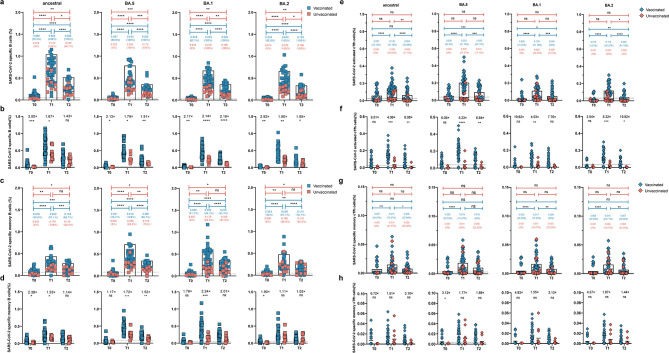
BA.5 infection enhanced cross-reactive spike-specific B cell responses and circulating follicular helper T (cTfh) cells that persist up to 5 months in vaccinated individuals. (a, c, e, and g) The dynamic frequency of spike-specific B cells (a), memory B cells (c), activated cTfh cells (e), and memory cTfh cells (g) specific to ancestral spike, BA.1 spike, BA.2 spike, and BA.5 spike at T0, T1, and T2 time points. Values above the symbols denote the median, and the percentage of positive responders was also noted. (b, d, f, and h) The comparison of SARS-CoV-2-specific B cells (b), memory B cells (d), activated cTfh cells (f), and memory cTfh cells (h) between vaccinated and unvaccinated individuals at T0, T1, and T2 time points. Fold change of spike-specific B cells between two groups at three time points. Data are shown as the fold change between vaccinated vs unvaccinated donors. Dotted lines indicate the limit of detection for the assay. Single comparison variables between two groups were performed using the Mann-Whitney *U* test. Multiple comparisons of specific memory B cell responses at three time points were performed using the Friedman’s one-way ANOVA with LSD. *P < 0.05; **P < 0.01; ***P < 0.001; ****P < 0.0001; and ns, no significant difference.

Beyond humoral immunity, cellular components are also particularly indispensable for long-term protection against COVID-19 (15). We have shown previously that while antibodies decline over time, B cells persist and cross-react with SARS-CoV-2 variants of concern to some degree (4, 5). Therefore, we evaluated the magnitude and cross-reactivity of the antigen-specific B cell response via flow cytometric enumeration of B cells stained with differentially labeled ancestral, BA.1, BA.2, and BA.5 spike proteins (Fig. 3a). At baseline, ancestral and BA.5 spike reactive B cells were detectable in 23 (79.3%) and 17 (58.6%) of 29 participants, comprising a median of 0.090% (95% CI 0.049%–0.125%) and 0.047% (95% CI 0.024%–0.061%) of the total B cells, respectively, among vaccinated individuals. Minimal ancestral spike-specific B cells (0.030%, 95% CI 0.020%–0.036%) were detected in unvaccinated controls (Fig. 3a). Correspondingly, BA.5 spike-specific B cells increased from 0.047% of the total B cells at baseline to 0.430% at 2 weeks and slightly decreased to 0.220% at 20 weeks after BA.5 infection. In contrast, BA.5-infected unvaccinated individuals had significantly lower levels of ancestral (1.67-fold lower, *P* = 0.018), BA.5 (1.79-fold lower, *P* = 0.002), and BA.1 (2.14-fold lower, *P* < 0.0001) spike-specific B cells at T1 compared with vaccinated individuals (Fig. 3b). Twenty weeks after BA.5 infection, unvaccinated individuals still exhibited a lower proportion of spike-reactive B cells than the vaccinated group across the tested Omicron subvariants, including BA.5 (1.91-fold lower, *P* = 0.007), BA.1 (2.19-fold lower, *P* < 0.0001), and BA.2 (1.95-fold lower, *P* = 0.014).

The proportion of spike-specific memory B cells among the total memory B cells was also analyzed (Fig. 3c). Triple-dose CoronaVac recipients showed a median of 0.076%, 0.048%, 0.055%, and 0.041% memory B cells specific to ancestral, BA.1, BA.2, and BA.5, respectively, even at 12 months after the third booster vaccination. Memory B cells were absent in unvaccinated individuals at T0. BA.5 breakthrough infection expanded cross-reactive spike-specific memory B cells. At T1, compared to unvaccinated controls, vaccinated individuals showed remarkably higher levels of memory B cells specific to the ancestral (0.170% vs 0.260%, 1.53-fold lower, *P* = 0.030), BA.5 (0.250% vs 0.430%, 1.72-fold lower, *P* = 0.0009), BA.1 (0.170% vs 0.380%, 2.24-fold lower, *P* = 0.0003), and BA.2 (0.180% vs 0.200%, *P* = 0.451) strains (Fig. 3c and d). At T2, vaccinated individuals still maintained higher levels of spike-specific memory B cells than unvaccinated individuals, but the difference was not statistically different except for BA.5-specific memory B cells. Collectively, our data indicate that triple-dose CoronaVac established spike-specific memory B cells that could rapidly expand and proliferate into antibody-producing B cells upon breakthrough infection.

### Vaccination enabled activation of circulating follicular helper T cells and expansion of memory cTfh cells upon BA.5 breakthrough infection

Tfh cells play a vital role in the selection and activation of B cells into activated antibody-secreting cells at germinal centers (GCs), which is critical for the development of long-lasting, high-affinity antibody responses (16). A small population of circulating follicular helper T (cTfh) cells resemble GC Tfh cells in terms of their phenotype and serve as a counterpart to GC Tfh cells to support antibody secretion (17). We tracked and depicted the frequency of spike-specific cTfh cells (Fig. 3e through h). BA.5 infection expanded ancestral-specific cTfh cells from 0.057% (0.033%–0.081%) at baseline to 0.139% (0.110%–0.169%) at T1, and BA.5 spike-specific cTfh cells from 0.050% (0.030%–0.070%) at baseline to 0.203% (0.163%–0.242%) at T1, among vaccinated individuals (Fig. 3e). The frequency of spike-specific cTfh cells across SARS-CoV-2 variants was 2.04–19.82-fold higher in vaccinated individuals than in unvaccinated individuals. In particular, the percentage of BA.5-specific cTfh cells in the vaccinated group was 0.203% (0.163%–0.242%) and 0.093% (0.067%–0.120%) at T1 and T2, respectively (5.23-fold and 6.84-fold higher than in unvaccinated individuals) (Fig. 3f).

At T0, despite being 12 months after the third dose of CoronaVac, vaccinated individuals not only showed a detectable level of ancestral spike-specific memory cTfh cell responses (0.007%, 95% CI 0.003%–0.011%) but they also showed a slightly lower frequency of memory cTfh cells that were cross-reactive to the BA.1, BA.2, and BA.5 spikes, ranging from 0.004% to 0.005% (Fig. 3g). There was a trend toward slightly higher spike-specific memory cTfh cells in vaccinated individuals than in unvaccinated individuals, but no significant difference was observed (Fig. 3h). Collectively, our data suggest that breakthrough infection could quickly recall and activate specific cTfh cells, but memory cTfh cells were not significantly elevated compared with unvaccinated individuals.

### BA.5 infection enhanced cross-reactive memory T cells among the recipients of triple-dose CoronaVac

**Fig 4:**
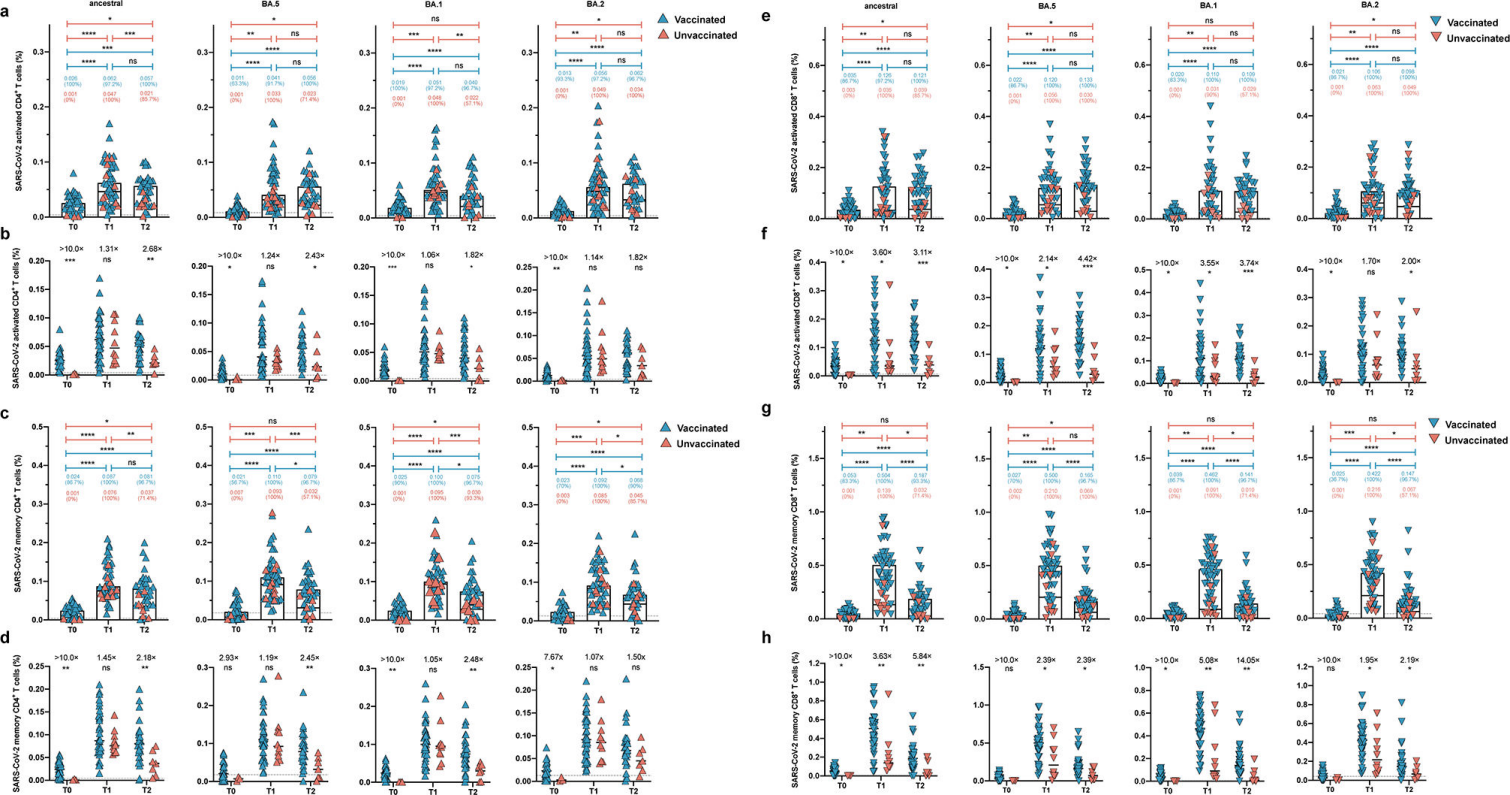
BA.5 infection enhanced cross-reactive memory T cells among previously triple CoronaVac vaccinees. (a, c, e, and g) The dynamic change of activated CD4+ T cells (a), memory CD4+ T cells (c), activated CD8+ T cells (e), and memory CD8+ T cells (g) specific to ancestral, BA.1, BA.2, and BA.5 spike in vaccinated and unvaccinated groups at T0, T1, and T2 time points. Values above the symbols denote the median, and the percentage of positive responders was also noted. (b, d, f, and h) The comparison of SARS-CoV-2-specific activated CD4+ T cells (b), memory CD4+ T cells (d), activated CD8+ T cells (f), and memory CD8+ T cells (h) between vaccinated and unvaccinated individuals at T0, T1, and T2 time points. Data are shown as the fold change between vaccinated vs unvaccinated donors. Single comparison variables between groups were performed using the Mann-Whitney *U* test. Multiple comparisons of specific T cell responses of three time points were performed using the Friedman’s one-way ANOVA with LSD. Dotted lines indicate the limit of detection for the assay. Bars represent the median value. *P* < 0.05 was considered to be statistically significant. *P < 0.05; **P < 0.01; ***P < 0.001; ****P < 0.0001; and ns, no significant difference.

T cells contribute to the defense against viral infections by orchestrating antibody production and cytotoxic killing of infected cells (18). Previously, we observed that CoronaVac induced durable, cross-reactive T cell responses (4, 5). Given their importance, we examined whether BA.5 breakthrough infection could quickly recall and expand ancestral- and BA.5-specific CD4^+^ and CD8^+^ T cell responses. The data show that triple-dose CoronaVac recipients still showed detectable CD4^+^ T cells with a median frequency of 0.026% (95% CI 0.016%–0.039%) (Fig. 4a) and CD8^+^ T cells with a median frequency of 0.035% (95% CI 0.019%–0.056%) (Fig. 4b). In contrast, few spike-specific CD4^+^ or CD8^+^ T cell responses were detected in unvaccinated individuals. At T1, BA.5 breakthrough infection effectively expanded both CD4^+^ T cell (3.73-fold, *P* < 0.0001) and CD8^+^ T cell (5.45-fold, *P* < 0.0001) responses specific to the BA.5 spike (Fig. 4a and e). Similar trends were also observed in the T cell responses across the ancestral strain and the Omicron subvariants at T2.

The memory T cell subpopulation is critical to determine anti-viral responses upon antigen exposure. Therefore, we longitudinally measured the frequency of specific memory T cell responses. We found that compared with the corresponding baseline samples, BA.5 breakthrough infection led to augmented BA.5 spike-specific memory CD4^+^ T cell (5.24-fold, *P* < 0.0001) and memory CD8^+^ T cell (18.52-fold, *P* < 0.0001) responses at T1 (Fig. 4c and g). Interestingly, there was a trend toward a slightly higher frequency of specific memory CD4^+^ T cell responses in vaccinated individuals than in unvaccinated controls, but without significant differences (Fig. 4d). In contrast, compared with unvaccinated controls, vaccinated individuals showed substantially higher levels of ancestral (0.504% vs 0.139%, *P* = 0.001), BA.5 (0.500% vs 0.210%, *P* = 0.011), BA.1 (0.462% vs 0.091%, *P* = 0.002), and BA.2 (0.422% vs 0.216%, *P* = 0.040) spike-specific CD8^+^ memory T cell responses (Fig. 4g and h). To summarize, CD4^+^ and CD8^+^ memory T cells were still detectable 12 months after the third dose of CoronaVac. These pre-existing cross-reactive T cell responses and memory subsets could be effectively boosted and maintained by BA.5 infection.

### The increase in BA.5-specific immune responses after breakthrough infection was consistently higher than for the ancestral strain

**Fig 5:**
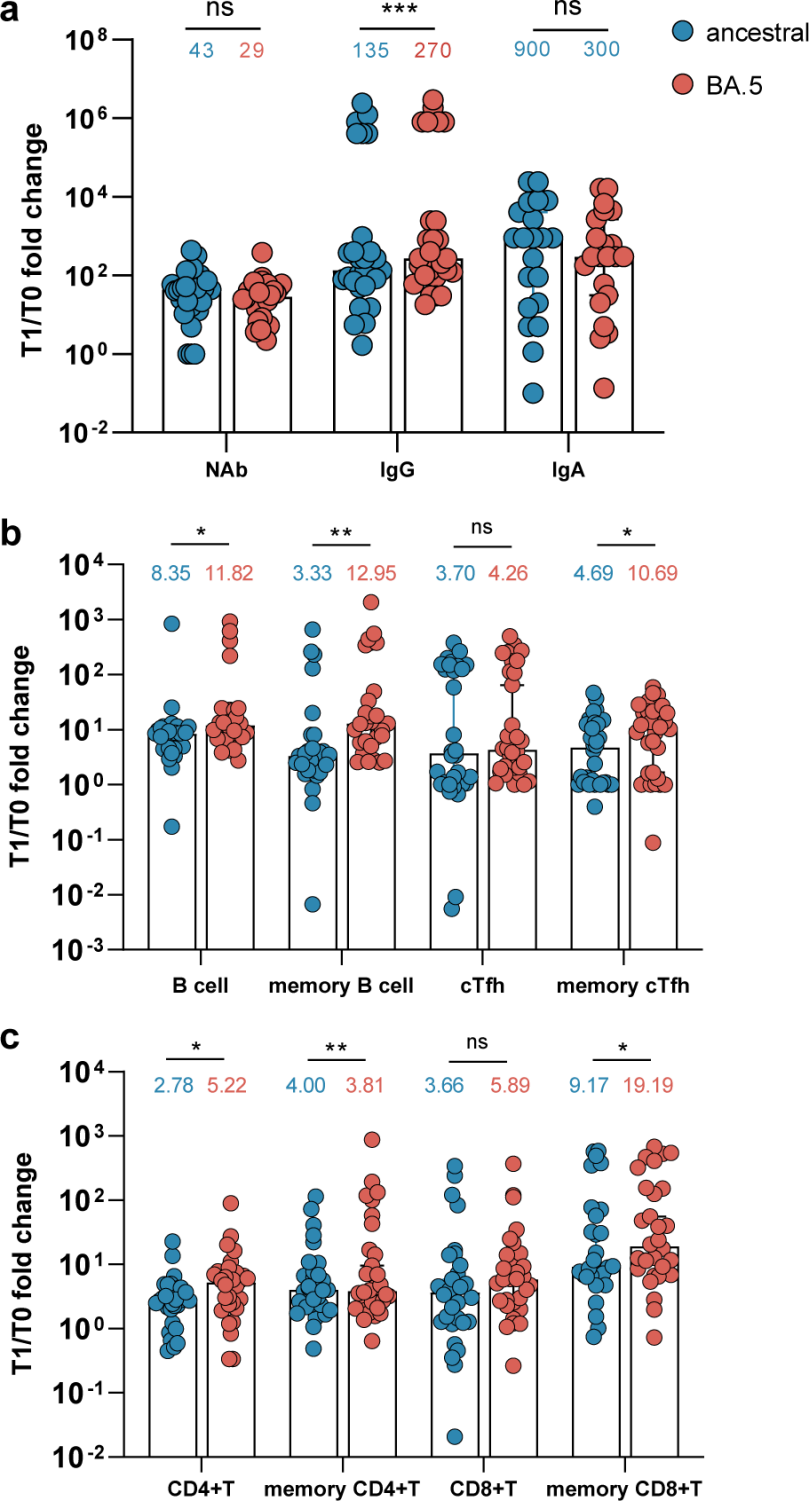
Increase in the fold of BA.5-specific immune responses post-breakthrough infection was consistently higher than those of ancestral strain. Fold change of serum neutralizing antibody responses (NAb), anti-spike IgG and IgA (**a**), B cells, memory B cells, cTfh and memory cTfh cells (**b**), CD4^+^ T cells, memory CD4^+^ T cells, CD8^+^ T cells, and memory CD8^+^ T cells (**c**) among vaccinated group against ancestral and BA.5 strain from T1 time point vs T0 time point. Data are shown as the fold change between T0 and T1 in the vaccinated group. Wilcoxon matched-pairs signed rank test was performed for paired data. *P < 0.05; **P < 0.01; ***P < 0.001; ****P < 0.0001; and ns, no significant difference.

Immune imprinting induced by ancestral strain-based vaccination might compromise the antibody response to Omicron-based boosters (8, 9). Therefore, we compared the increases in the ancestral and BA.5-specific immune responses in vaccinated individuals (Fig. 5). Notably, we found that the increase from T0 to T1 in serum neutralization activities specific to BA.5 was comparable to that for the ancestral strain, while the enhancement from T0 to T1 in the BA.5 spike-specific IgG response was substantially higher than for the ancestral spike (Fig. 5a). Similarly, the majority of BA.5-specific immune parameters, including B cells, memory B cells, memory cTfh cells (Fig. 5b), CD4^+^ T cells, memory CD4^+^ T cells, and memory CD8^+^ T cells (Fig. 5c), displayed substantially greater fold changes than those specific to the ancestral strain at T1.

### Distinct immune trajectories in breakthrough-infected individuals vs naturally infected individuals

**Fig 6:**
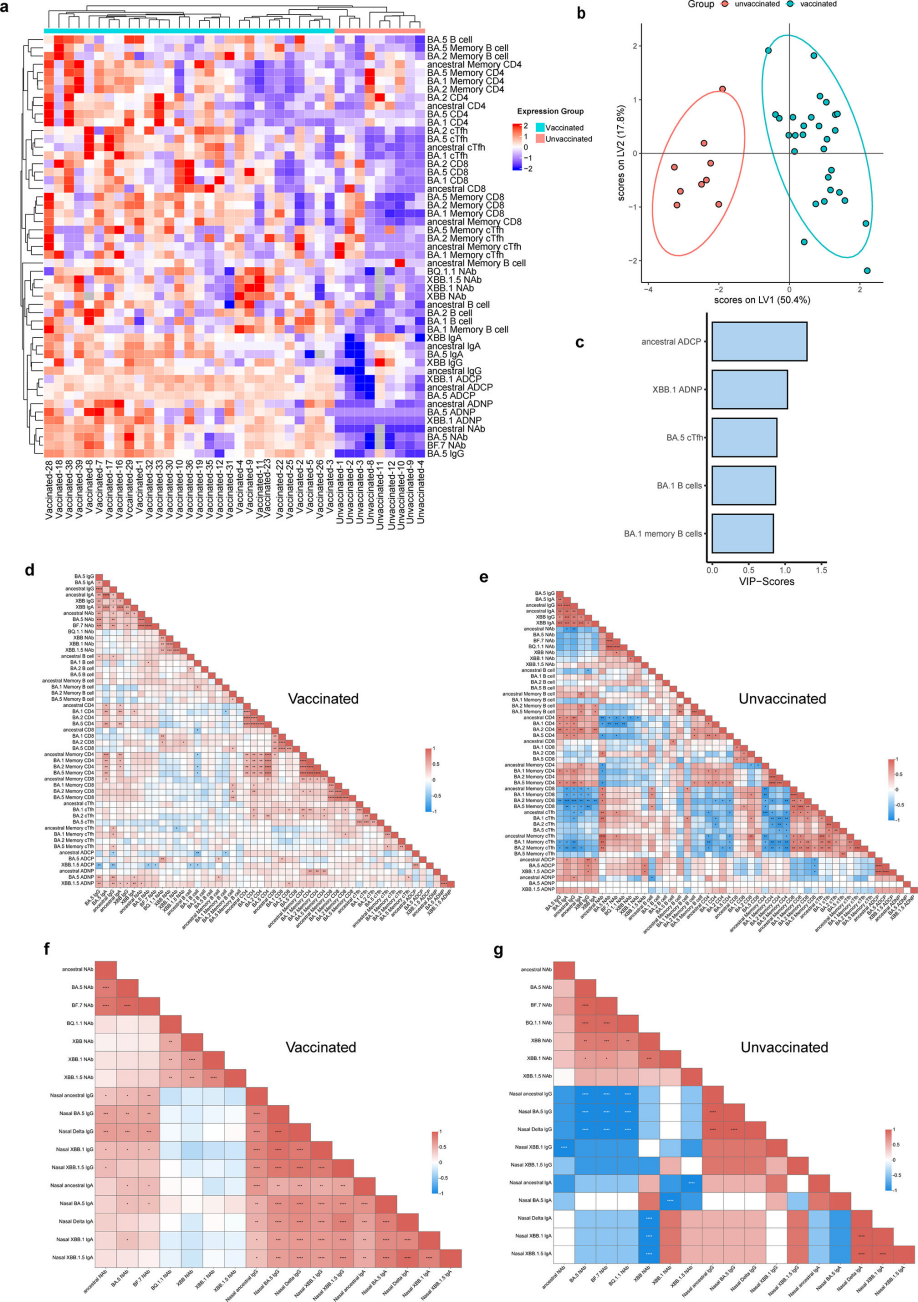
Distinct patterns of immune trajectories in breakthrough-infected individuals vs naturally infected donors. (**a**) A cold-to-hot heatmap and hierarchical clustering represent the scaled magnitude of SARS-CoV-2-specific humoral, mucosal, and cellular immune responses, including spike-binding antibody, neutralization activities, Fc-mediated effector function, cellular responses, at T1 time point after BA.5 infection between vaccinated vs unvaccinated group. Each column represents vaccinated or unvaccinated individuals, while each role represents an immune parameter. The distribution of markers and participants in the cohort was automatically performed by supervised hierarchical clustering. (**b**) A Least Absolute Shrinkage Selection Operator (LASSO) was used to select antibody features that contributed most of the discriminate vaccinated vs unvaccinated individuals. A partial least square discriminant analysis was used to visualize samples. LASSO-selected features were ranked based on their variable importance in projection (VIP) score, and the loadings of the latent variable 1 (LV1) were visualized in a bar graph. (c) Selected immune parameter features were ordered according to their VIP score. (**d and e**) Correlation analysis between the parameters of adaptive immune responses, including binding antibody, neutralization activities, B cell, CD4 T cell, CD8 T cell, cTfh cell responses as well as their memory subsets at T1 time point after BA.5 infection in vaccinated (**d**) vs unvaccinated donors (**e**). (**f and g**) Correlation analysis of mucosal antibody response and serum neutralization activity in vaccinated individuals (**f**) vs unvaccinated (**g**) donors. The strength of a correlation (Sperman’s correlation coefficient) is demonstrated by the color of the square, and the significance is indicated by asterisks. Correlation between two continuous variables was analyzed using the Spearman correlation analysis. *P < 0.05; **P < 0.01; ***P < 0.001; ****P < 0.0001; and ns, no significant difference.

The BA.5-infected vaccinated group exhibited remarkable divergence in SARS-CoV-2-specific immune responses compared with the unvaccinated group. Therefore, further exploratory analyses were performed to gain a more detailed understanding of their fundamental differences. Following centering and scaling, hierarchical clustering of the participants revealed that vaccinated samples clustered away from the unvaccinated samples (Fig. 6a), highlighting the potent multicomponent immune memory induced by CoronaVac, even up to 12 months after the third CoronaVac dose. A multivariate partial least squares-discriminant analysis was performed across the vaccinated group and the unvaccinated group at the peak immune response after breakthrough infection (Fig. 6b). We found that vaccinated individuals could be perfectly separated from unvaccinated individuals. Notably, the immune profiles of participants in the vaccinated group exhibited selective enrichment of ancestral-specific ADCP, XBB.1-specific ADNP, BA.5-specific cTfh cells, and BA.1-specific B cells and memory B cells (Fig. 6c).

We also performed correlation analyses for the measured immune parameters, including antibody binding, neutralization activity, B cells, CD4^+^ T cells, CD8^+^ T cells, and cTfh cell responses, as well as their memory subsets, among the vaccinated group and the unvaccinated group. In the vaccinated group, we observed a moderate-to-low degree of correlation among the parameters defining the antibody response and among the parameters defining T cell responses. Furthermore, binding antibody responses were positively correlated with the CD4^+^ T cell response and the corresponding memory subsets (Fig. 6d). In the unvaccinated group, we observed a strong or moderate degree of correlation within the antibody compartment and within the T cell compartment. Nevertheless, a considerable proportion of inverse correlations were identified between antibodies and CD8^+^ memory T cells and cTfh cells, as well as between the CD4^+^ T cell response and memory cTfh cells (Fig. 6e). These results suggest that breakthrough infection had a well-integrated adaptive response between the antibody compartment and the T cell compartment, whereas the unvaccinated group harbored less coordinated immune trajectories between the antibody and T cell compartments, which might contribute to suboptimal antibody responses.

Additionally, we investigated the correlation between systemic and mucosal humoral immunity. Interestingly, BA.5-infected individuals in the vaccinated group demonstrated a significant positive correlation between serum-neutralizing activity and nasal IgA binding as well as IgG responses. Furthermore, strong positive correlations were identified within nasal IgA and IgG binding specific to SARS-CoV-2 variant spike antigens (Fig. 6f). Surprisingly, for previously unvaccinated individuals, serum-neutralizing activity was negatively correlated with nasal-binding antibody titers after BA.5 infection. Additionally, few cross-correlations were identified among the parameters of the nasal IgG- and IgA-binding antibodies (Fig. 6g). Collectively, our data highlight that vaccinated individuals have better synchronization of multiple immune components than unvaccinated individuals upon heterologous SARS-CoV-2 infection.

## DISCUSSION

The inactivated whole-virion vaccine CoronaVac is one of the most widely used COVID-19 vaccines worldwide (19). Although there has been continual evolution of viral variants, which have managed to evade antibody responses to varying degrees, triple-dose CoronaVac has retained more effectiveness against severe disease than against overall infection (20). Emerging evidence suggests that both T cells and antibody responses provide the greatest protection against infection and death in severe COVID-19 cases (21). In this study, we provide evidence of immune memory responses to ancestral and Omicron subvariants after BA.5 breakthrough infection among triple-dose CoronaVac recipients, which are maintained 12 months after the last dose. We also performed head-to-head comparisons of immune responses among vaccinated individuals vs unvaccinated individuals. When considered alongside the risk of severe infection and the long-term consequences of infection, our findings have important implications for vaccine policy.

Understanding the durability of the protection conferred by SARS-CoV-2 vaccination is of utmost importance to guide COVID-19 mitigation policies worldwide. The first key question we addressed is the durability of the immune memory and whether it is likely to protect against severe COVID-19 in the long term. We showed that serum anti-spike IgG and IgA responses were still detectable among the majority of triple-dose CoronaVac recipients at 12 months after the last dose. Additionally, we found that B and T cell responses after triple-dose CoronaVac were durable up to 12 months after the third dose, and that ancestral and Omicron subvariants were equally well recognized. Taken together, our longitudinal follow up over 12 months after the third CoronaVac dose demonstrated the durability and long-term maintenance of SARS-CoV-2 circulating B cells and CD4^+^ and CD8^+^ memory T cells.

The second key question that we attempted to address is the impact of immune imprinting from triple-dose CoronaVac on the adaptive response against Omicron subvariants. Our study analyzed breakthrough infection-induced humoral responses and cellular responses simultaneously, which allowed us to profile the dynamic kinetics of the antibody and cellular compartments. As the majority of people are vaccinated against the ancestral strain, immune imprinting induced by ancestral strain-based vaccination presents a major challenge to the performance of updated boosters. It has been suggested that boosting with a variant that is antigenically distinct from the ancestral strain would recall pre-existing memory B cells induced by the ancestral strain, preventing the generation of humoral immune responses targeting new variants (9, 22). Intriguingly, our results did not show evidence of antigenic sin in the antibody responses after Omicron infection. We speculate this discrepancy might be largely due to the relatively longer interval (12 months) between breakthrough infection and the third CoronaVac (9, 23, 24). The long interval before Omicron breakthrough infection might preferentially recall cross-reactive antibodies that exhibit increased affinity and neutralization potency against Omicron, which could then drive a greater response given a subsequent antigen exposure (25). In addition, our finding aligns with recent evidence showing that either Omicron vaccination boosting or breakthrough infection not only triggers rapid memory cellular responses but also induces *de novo* B (26) and T cell (27) responses. Furthermore, we also compared the immune imprinting on T cell responses, which are less affected by viral variants than antibodies. This is likely due to the broader range of epitopes available to T cells compared with antibodies, where protective responses are more focused. Consistent with our findings, clinical and epidemiological studies also support our notion that any prior exposure to SARS-CoV-2 by infection and/or vaccination has a positive impact in mitigating the disease severity of subsequent infections (28).

Further support for the protective role of triple-dose CoronaVac comes from our observation that hybrid immunity strengthens and broadens antibody responses, mucosal immunity, and cellular immunity, providing the most sustained protection. Despite there being a large amount of evidence highlighting the remarkable enhancement of serological and cellular responses elicited by hybrid immunity, there is a paucity of data suggesting that mucosal immunity could also be strengthened. For the first time, our data demonstrate that a larger proportion of breakthrough-infected individuals harbored respiratory spike-specific IgA responses than naturally infected individuals. Additionally, spike-specific nasal IgG and IgA responses of remarkable magnitude were also identified in individuals with hybrid immunity. The augmented mucosal immunity, especially the nasal IgA response (29), might be strongly associated with superior protection against SARS-CoV-2 reinfection, as described previously (30).

Our study has important implications for the vulnerability of unvaccinated Omicron-infected individuals to reinfection by circulating and emerging SARS-CoV-2. In the absence of vaccination, BA.5 infection-elicited humoral responses showed remarkably reduced serum neutralization activity against BA.5 and BF.7, as well as a subtle decrease against BQ1.1, XBB, XBB.1, and XBB.1.5, and dramatic loss against the ancestral strain. Additionally, despite infection via the respiratory route, the BA.5 spike nasal IgA and IgG titers were dramatically lower in unvaccinated controls than in vaccinated individuals after BA.5 infection, indicating the possible risk of reinfection. We extended our analysis to Fc-mediated phagocytosis and mucosal immunity. In particular, we found that BA.5-specific ADNP responses and nasal IgA responses were not effectively induced in unvaccinated individuals after BA.5 infection. In contrast, the BA.5-specific T cell response was only subtly lower in unvaccinated individuals than in vaccinated individuals. Taken together, the modest immune responses in BA.5-infected unvaccinated individuals leave this unvaccinated group at risk of being reinfected with Omicron subvariants. Our data indicate that Omicron-based vaccines might not be an ideal immunogen in SARS-CoV-2-naive individuals.

For the first time, our results provide an immune landscape of hybrid immunity elicited by BA.5 infection in triple-vaccinated vs unvaccinated individuals. We showed that vaccination with triple-dose CoronaVac effectively evoked higher-quality immune responses characterized by convergent development of cross-reactive humoral and cellular immune compartments, as well as collaborative systemic and mucosal antibody compartments. Machine learning analysis showed that vaccinated individuals generated potent immune responses involving ADCP, ADNP, cTfh cells, activated B cells, and memory B cells, with distinct patterns from unvaccinated donors. Our data also suggest that a one-time Omicron BA.5 infection might not be sufficient to trigger cross-reactive humoral and cellular responses.

This study has some limitations that should be noted. First, T and B cells in the peripheral blood only account for a small proportion of the T and B cell population in the body. Therefore, the measurement of circulating cellular responses might not fully represent the landscape of T cell immunity in vaccinated individuals, especially tissue-resident memory T cells. Second, the participants in our cohort were generally young; older individuals were not included. Third, we used peptide stimulation to quantitatively measure T and B cell responses to SARS-CoV-2 spike proteins by flow cytometry. The use of conjugated peptide major histocompatibility complex multimers might result in the sensitive detection of antigen-specific T cells at a higher resolution. Finally, analysis of a more diverse cohort might facilitate further dissection of the immune responses induced by other types of vaccination.

In summary, we have shown that triple-dose CoronaVac remarkably augments antibody, mucosal, and cellular responses that are potent, durable, and cross-reactive to Omicron subvariants. This study sheds light on the dynamics of human adaptive immunity to SARS-CoV-2 Omicron subvariants in a highly vaccinated population with inactivated COVID-19 vaccines.

## MATERIALS AND METHODS

### Study design

The purpose of this prospective observational study (NCT05680896) was to directly compare the humoral and cellular immune responses among triple CoronaVac-vaccinated individuals or unvaccinated individuals who have subsequently contracted the Omicron BA.5 infection during the Omicron wave last December in China. Serum samples were collected from the participants, which were analyzed using ELISA, pseudovirus neutralization assay, antibody-dependent cellular phagocytosis, as well as flow-cytometry-based cellular analysis.

### Cohort selection and sample collection

Healthcare workers at Nanjing Drum Tower Hospital were recruited and enrolled in the study belonging to two groups: vaccinated and unvaccinated individuals. The vaccinated group received the first two doses of CoronaVac in February 2021 and the third dose of CoronaVac in November 2022, while the unvaccinated individuals did not receive any COVID-19 vaccine prior to Omicron BA.5 infection. Both groups had PCR-confirmed diagnosis of COVID-19 during the recent Omicron wave from December 2022 and January 2023, and the infected viral sequence was further confirmed by next-generation sequencing. For vaccinated participants, baseline samples were collected at 12 months after the third CoronaVac immunization. The post-infection samples were collected at the median of 18 days (15–21) and 146.5 days (144.8–150.0) post-BA.5 infection in the vaccinated group, and 14 days (13.5–15) and 127.5 days (113.8–140.3) in the unvaccinated group, respectively.

Sera were obtained by collecting 4–6 mL of whole blood in a BD Vacutainer Plus Plastic Serum Tube, which was centrifuged for 10 min at 1,000 × *g* before serum was aliquoted and stored at −20°C. Meanwhile, the nasal swabs were also collected for the measurement of specific mucosal antibody titers. For the cellular analysis, peripheral blood mononuclear cells (PBMC) were isolated from the blood collected in ethylenediaminetetraacetic acid-anticoagulated tubes by lymphocyte separation medium density gradients (Stemcell Technologies, cat# 07801) and resuspended in PRMI 1640 medium supplemented with 10% fetal calf serum (FCS), 1% penicillin/streptomycin, and 1.5% HEPES buffer (complete medium; Thermo Scientific) for stimulation assays or stored at −135°C until used.

### Protein and peptides

Pools of 15-mer peptides overlapping by 11 amino acids (aa) and together spanning the entire sequence of SARS-CoV-2 spike glycoprotein (S) from ancestral, Omicron BA.1 (B.1.1.529), BA.2 (B.1.1.529.2), and BA.5 (B.1.1.529.5) variants were used for *ex vivo* stimulation of PBMC for flow cytometry. The ectodomain of ancestral SARS-CoV-2 spike (GenBank: MN908947.3) was expressed as previously described (31). The protein was purified from FreeStyle 293-F cells (Thermo Fisher Scientific, USA) using affinity chromatography followed by size exclusion chromatography, detailed as described previously (31).

### Measurement of serum and nasal SARS-CoV-2 spike-specific humoral responses

Serum or nasal swabs for immunological assessments were taken at three different time points, including 12 months after the third dose before BA.5 infection, 2 weeks, and 20 weeks post-BA.5 infection (4, 31). Antigen-specific serological and nasal swab antibodies against SARS-CoV-2 were determined by an in-house enzyme-linked immunosorbent assay, as previously described. Antibody-dependent cellular phagocytosis and antibody-dependent neutrophil phagocytosis (ADNP) were described in the previous study (7). The pseudovirus neutralization assay was performed using lentivirus-based SARS-CoV-2 pseudoviruses, which were provided by Vazyme Biotech Co., Ltd, as previously described (4, 32).

### Antigen-specific measurement of cellular analyses

The biotinylated ectodomain of spike protein was fluorescently labeled to identify SARS-CoV-2-specific circulating B cells and memory B cells. The detailed approach has been described in previous studies (4, 5). To measure antigen-specific circulating and memory CD4^+^, CD8^+^ T cells, and cTfh cells, an activation-induced marker assay was performed as described previously (4, 5). Stained samples were acquired on a fluorescence-activated cell sorter FACSAria III Cell Sorter instrument (BD Biosciences) and analyzed using FlowJo software version 10.7.1 (FlowJo LLC, BD Bioscience).

### Data analysis

To show the potential distinction of immune parameters between two groups, a cold-to-hot hierarchical clustering heatmap represents the scaled magnitude of SARS-CoV-2-specific humoral, mucosal, and cellular immune responses, including spike-binding antibody titers, neutralization activities, Fc-mediated effector function, cellular responses, at T1 time point between vaccinated vs unvaccinated group. Each column represents a vaccinated or unvaccinated individual, while each row represents an immune parameter. The distribution of markers and participants in the cohort was automatically performed by supervised hierarchical clustering. ComplexHeatmap R package (2.15.1) was used for analysis.

A multivariate classification model was built to discriminate immunological profiles among triple vaccinated and unvaccinated groups using tested adaptive immune parameters in our study. All data were normalized using *z*-scoring before analysis. Feature selection was performed using the least absolute shrinkage and selection operator. Partial least square discriminant analysis (PLS-DA) was used for the classification and visualization of immune parameters from two groups. Selected immune features were ordered based on variable importance in projection score, and the first two latent variables of the PLS-DA model were used for visualization. R package “ropls” version 1.20.043, “glmnet” version 4.0.244, and the systemsseRology R package (v.1.1) (https://github.com/LoosC/systemsseRology) were used for analysis.

For the cross-correlation analysis, Spearman correlation analysis was used for correlation analysis between all tested immune parameters. The correlation analysis was presented using ChiPlot (https://www.chiplot.online/correlation_heatmap.html).

### Statistical analysis

Binding antibody titers or neutralizing titers were expressed as geometric mean titers. The mean (standard deviation) or median (95% confidence interval) was used to present the continuous variables. Categorical variables were described as counts and percentages. Single comparison variables between groups were performed using the Mann-Whitney *U* test. Multiple comparisons of antibody titers, specific activated or memory B cell and T cell responses were performed using the Friedman’s one-way ANOVA with least significant difference (LSD). Correlation between two continuous variables was analyzed using the Spearman correlation analysis. *P* < 0.05 was considered to be statistically significant.*P < 0.05; **P < 0.01; ***P < 0.001; ****P < 0.0001; and ns, no significant difference. SPSS software program version 22.0 (Chicago, IL, USA) was used for data analysis.

## Data Availability

R package "ropls" version 1.20.043 and "glmnet" version 4.0.244 and the systemsseRology R package (v.1.1) (https://github.com/LoosC/systemsseRology) were used for analysis. The correlation analysis was presented using ChiPlot (https://www.chiplot.online/correlation_heatmap.html).

## SUPPLEMENTAL MATERIAL

Supplementary Figures mbio.02407-23-s0001.doc Figures S1-S4.

ASM does not own the copyrights to Supplemental Material that may be linked to, or accessed through, an article. The authors have granted ASM a non-exclusive, world-wide license to publish the Supplemental Material files. Please contact the corresponding author directly for reuse.
