# Supplementary material for: SARS-CoV-2 Omicron infection augments the magnitude and durability of systemic and mucosal immunity in triple-dose CoronaVac recipients
Source: mBio. 2024 Mar 8;15(4):e02407-23. doi: 10.1128/mbio.02407-23 (PMC11005357; doi:10.1128/mbio.02407-23)

**Supplementary figures**

**Figure S1. Flow cytometry gating strategies to define SARS-CoV-2 RBD-specific B cells. (a)** Lymphocytes were gated based on forward- and side-scatter and single cells were gated based on SSC-A and SSC-H. Total B cells were identified as CD19^+^cells. Memory B cells were identified as CD38^-^CD27^+^cells. Spike specific B cells were identified based on binding to corresponding spike probes. (**b**) Representative samples between the vaccinated group and the unvaccinated group at T1 timepoint.


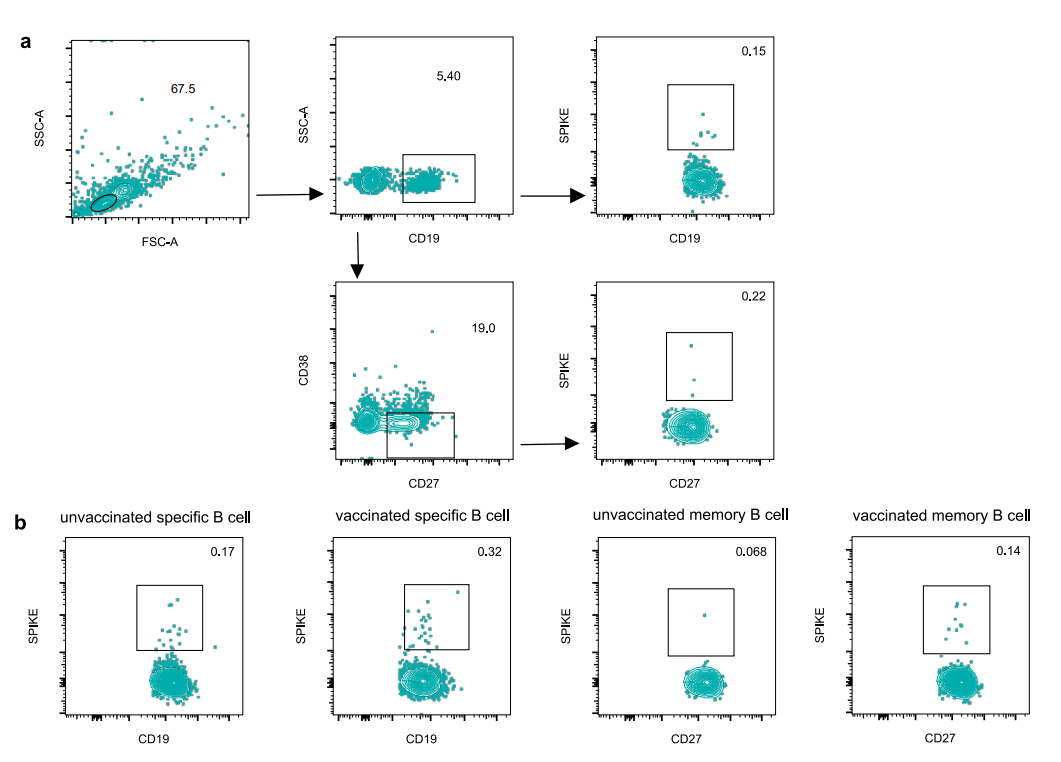


**Figure S2. Flow cytometry gating strategy for spike specific activated cTfh cells and memory cTfh cells. (a)** Representative flow cytometry gating strategy of spike specific activated cTfh cells and memory cTfh cells. **(b)** Representative flow cytometry gating strategy for activated cTfh cells and memory cTfh cells in the vaccinated group and non-vaccinated group.


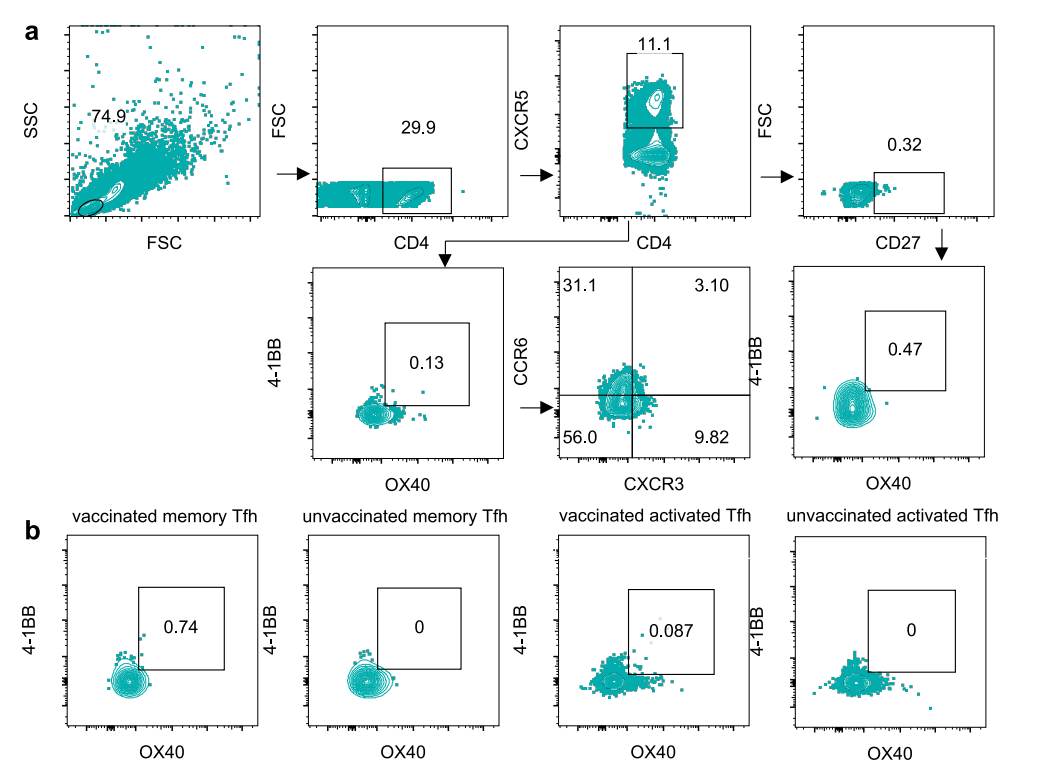


**Figure S3. Flow cytometry gating strategy for spike specific activated CD4+ T cells and memory CD4+ T cells.** (**a**) Representative flow cytometry gating strategy of spike specific activated CD4^+^ T cells and memory CD4^+^ T cells. (**b**) Representative flow cytometry gating strategy for activated CD4^+^ T cells and memory CD4^+^ T cells between the vaccinated group and non-vaccinated group.


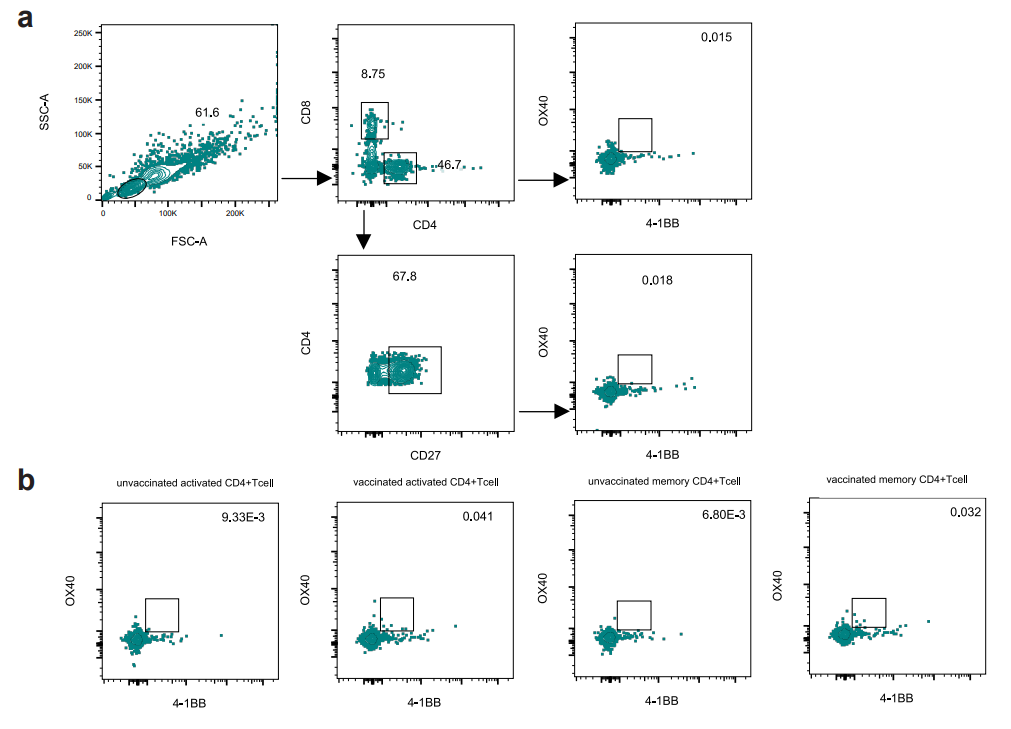


**Figure S4. Flow cytometry gating strategy for spike specific activated CD4^+^ T cells and memory CD8^+^ T cells.** (**a**) Representative flow cytometry gating strategy of spike specific activated CD8^+^ T cells and memory CD8^+^ T cells. (**b**) Representative flow cytometry gating for activated CD8^+^ T cells and memory CD8^+^ T cells between the vaccinated groups and non-vaccinated groups.


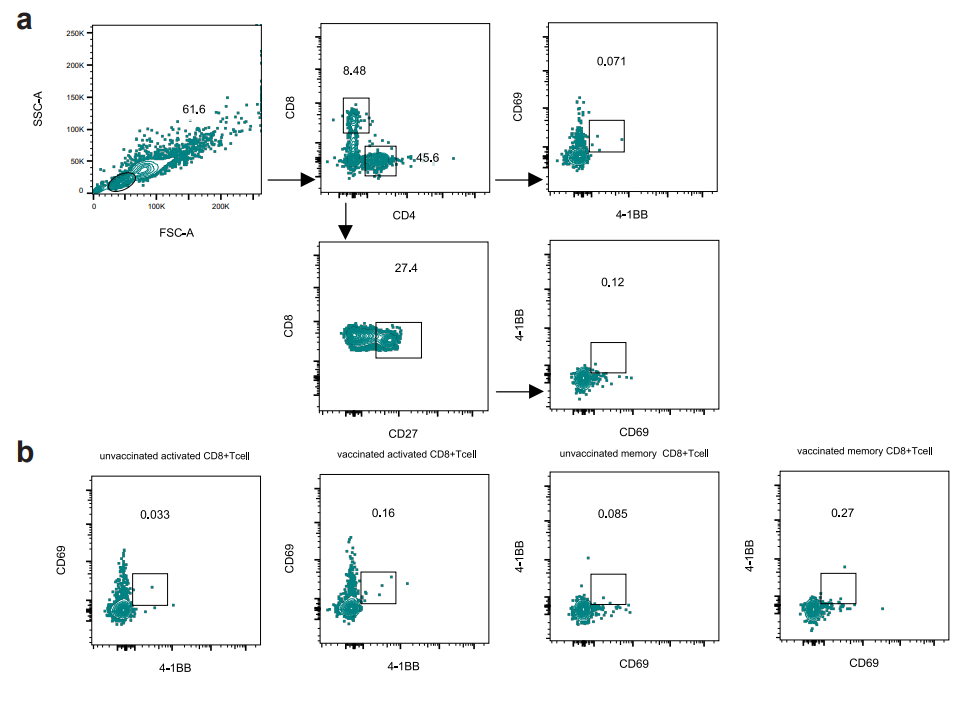

Supplement: Supplementary Figures — Figures S1-S4. [file mbio.02407-23-s0001.doc]
